# Metagenome-assembled bacterial genomes derived from various aging flue-cured tobacco samples

**DOI:** 10.1128/mra.00427-24

**Published:** 2024-07-22

**Authors:** Minghou Li, Yingjie Dong, Chen Liu, Haoran Liu, Zhen Wang, Jingru Ma, Xinhui Wang, Xiaojun Zhang

**Affiliations:** 1 State Key Laboratory of Microbial Metabolism, Shanghai Jiao Tong University, Shanghai, China; 2 Technology Center, Gansu Tobacco Industry Co.，Ltd., Lanzhou, Gansu Province, China; The University of Arizona, Tucson, Arizona, USA

**Keywords:** nicotine, degrading genes, tobacco, metagenome

## Abstract

We recovered 16 bacterial metagenome-assembled genomes from 11 flue-cured tobacco samples with different aging stage and various geographic origins. Their sizes range from 2.3 M to 5.4 M, with GC contents of 43.17%–74.45%, completeness of 78.80%–99.25%, and contamination of 0.47%–8.56%.

## ANNOUNCEMENT

Nicotine is the main alkaloid in tobacco (1). Besides its toxicity to humans (2 – 4), nicotine is also considered as a pollutant to the environment (5, 6). Here, 16 bacterial metagenome-assembled genomes (MAGs) from 11 different flue-cured tobaccos were retrieved. Six of them may be nicotine degradation genomes.

Fifteen fresh tobacco leaf samples were collected from Shanxi Province (A1, A2, B1, B2, and B3), Gansu Province (C1, C2, D1, and D2), and Yunnan Province (E1, E2, E3, E4, E5, and E6) in China in different years ranging from 2013 to 2022. After harvesting, the tobacco leaves were transported to Lanzhou City, Gansu Province, for their respective storage and aging (7). An Omega Bio-tek E.Z.N.A Bacterial DNA Kit was used to extract the DNA of 20-g dry leaves from above tobacco samples, five technical replicates for each type of sample. NEXTFLEX Rapid DNA-Seq Kit was used to create sequencing libraries, and paired-end (150 bp) reads were generated on Illumina NovaSeq platform (Illumina, San Diego, California, USA) by Shanghai Majorbio Bio-pharm Technology Co., Ltd. On average, 18,302,318,890 bp and 121,207,410 reads were sequenced for each library.

Unless otherwise noted, all software used default parameters. Adapter removal and quality trimming were done using fastp (v0.20.0) (8). Qualified reads that mapped to a genome of *Nicotiana* (GCF_001879085.1) were removed by Burrows-Wheeler Alignment tool (BWA) (v0.7.17) (9). Clean reads from five replicates of the same sample were mixed and assembled by MEGAHIT (v1.1.2) (10) with the option of min-contig-len 300. Prodigal (v2.6.3) (11) was used to predict the coding sequences in the contigs, and predicted genes were annotated by KOBAS (v2.0) (12). Metabat2 (v2.12.1) (13), CONCOCT (v0.5.0) (14), and Maxbin2 (v2.2.5) (15) were used for binning. DAS_Tool (v1.1.0) (16) was used to integrate three binning results to obtain an optimal, non-redundant set of bins. RefineM (v0.0.24) (17) was used to refine the bins. CheckM (v1.0.12) (18) was used to evaluate the completeness and contamination of each bin, and dRep (v3.4.2) (19) was used to remove the redundant bins. Taxonomic assignments of the non-redundant bins were performed by the GTDB-Tk (v2.3.0) (20). Gene models in each genome were identified using GeneMarkS (v4.28) (21) and annotated by KofamKOALA (v2024-03-01) (22).

As a result, a total of 16 bacterial MAGs were recovered from 11 samples since no MAGs met the quality cutoff in B3, C1, C2, and E6 samples. These MAGs have a completeness of >75% and a contamination of <10% (Table 1). Length varied from 2.3 to 5.4 Mb, with GC content ranging between 43.17% and 74.45%. Predicted gene annotation results showed that the nicotine-degrading genes, such as *ndhA*/*B*/*C* (23) and *NicA2* (24), were mainly affiliated with *Nocardioides* and *Pseudomonas*. It implies that six MAGs from these two genera might be nicotine degraders. However, no nicotine-degrading genes were found in these MAGs. The reason for degrading genes lost in the assembled genomes is probably due to the binning method, since the nicotine-degrading genes from *Nocardioides* and *Pseudomonas* had been reported to be located in the plasmids (1, 25).

**TABLE 1:** Accession numbers and summary of MAG properties

MAGs	Accession	Contigs num	Total length (bp)	N50 (bp)	GC content (%)	Completeness (%)	Contamination (%)	Strain heterogeneity (%)	Taxonomy	Potential function
A1_1	JBEFCT000000000	643	2336523	4916	44.08	78.80	5.27	61.11	*Acinetobacter soli*	
A1_3	JBEFCU000000000	199	4566799	56776	65.24	97.84	4.29	7.14	*Halomonas desiderata*	
A1_5	JBEFCV000000000	212	4939342	58383	53.66	85.50	7.76	57.14	*Dickeya zeae*	
A1_6	JBEFCW000000000	670	3516773	6667	74.39	91.63	1.69	40.00	*Nocardioides kribbensis*	Degrading nicotine
A2_2	JBEFDH000000000	558	5473796	14741	58.60	95.74	4.71	27.78	*Pseudomonas_E amygdali*	Degrading nicotine
B1_1	JBEFDI000000000	664	3521551	6991	74.45	91.94	1.74	75.00	*Nocardioides kribbensis*	Degrading nicotine
B2_3	JBEFCX000000000	651	3575302	7076	74.45	94.69	1.56	66.67	*Nocardioides kribbensis*	Degrading nicotine
D1_1	JBEFCY000000000	88	3111975	76036	43.17	99.25	0.56	50.00	*Enterococcus_D casseliflavus*	
D2_1	JBEFCZ000000000	1142	4228013	4550	68.89	81.49	8.56	26.32	*Methylobacterium extorquens*	
E1_1	JBEFDA000000000	1043	4366645	5626	55.59	91.95	5.37	93.75	*Pantoea agglomerans*	
E2_1	JBEFDB000000000	964	5479297	9708	59.02	91.02	4.83	48.94	*Pseudomonas_E amygdali*	Degrading nicotine
E3_1	JBEFDC000000000	1363	4115626	3747	55.87	84.84	7.18	84.91	*Pantoea agglomerans*	
E4_1	JBEFDD000000000	1053	4447229	6044	55.72	87.97	4.56	87.04	*Pantoea agglomerans*	
E4_2	JBEFDE000000000	641	3892505	8147	69.42	93.01	1.72	46.67	*Pseudomonas_S boreopolis*	Degrading nicotine
E5_1	JBEFDF000000000	525	3173955	7653	55.39	82.34	1.19	63.64	*Mixta sp940807255*	
E5_2	JBEFDG000000000	98	3425164	53969	61.45	98.89	0.47	100.00	*Corticimicrobacter sp*.	

## Data Availability

The raw reads were deposited at the Sequence Read Archive with accession numbers from SRR28810106 to SRR28810180 under the BioProject PRJNA1102117. All the accession numbers of assembled genomes in GeneBank are listed in Table 1.
